# Tension Pneumothorax and Subcutaneous Emphysema Complicating Insertion of Nasogastric Tube

**DOI:** 10.1155/2015/690742

**Published:** 2015-09-10

**Authors:** Narjis AL Saif, Adel Hammodi, M. Ali Al-Azem, Rasheed Al-Hubail

**Affiliations:** Critical Care Department, King Fahad Specialist Hospital, P.O. Box 15215, Dammam 31444, Saudi Arabia

## Abstract

Nasogastric tube has a key role in the management of substantial number of hospitalized patients particularly the critically ill. In spite of the apparent simple insertion technique, nasogastric tube placement has its serious perhaps fatal complications which need to be carefully assessed. Pulmonary misplacement and associated complications are commonplace during nasogastric tube procedure. We present a case of tension pneumothorax and massive surgical emphysema in critically ill ventilated patient due to inadvertent nasogastric tube insertion and also discussed the risk factors, complication list, and arrays of techniques for safer tube placement.

## 1. Introduction

Nasogastric tube insertion is a common procedure in hospitalized, particularly critically ill patients. Simple yet serious, this procedure may carry severe complications, increasing the odds of morbidity and mortality. The interactions between patient and procedure risk factors probably aggravate the range of drawbacks. Training, observation, and confirmation techniques would help to prevent or at least minimize the complication and maximize safe practice.

## 2. Case Report

### 2.1. History

Sixty-year-old male patient was known to have type II diabetes mellitus, hypertension, 4-year postrenal transplant, and hepatitis C cirrhosis. The patient was admitted to the hospital complaining of watery diarrhea that proved to be due to cytomegalovirus (CMV) colitis. During hospital stay, he developed respiratory distress and hypoxia, so he was transferred to the intensive care unit (ICU).

### 2.2. Assessment and ICU Course

The patient was intubated with no airway difficulty, connected to mechanical ventilation with SIMV/pressure support mode, FiO_2_ 0.4, and PEEP of 12 cmH_2_O to maintain oxygen saturation of 95%. His chest X-ray showed bilateral airspace disease that was suggestive of CMV pneumonitis. The patient was in a shock status requiring norepinephrine infusion of 18 micrograms/min. The patient was sedated with fentanyl and propofol targeting Richmond Agitation Sedation Scale (RASS) of −2.

In order to establish enteral feeding, attempts of insertion of nasogastric tube had failed. By the third attempt, a 16-French size, stylet-stiffened polyurethane nasogastric tube was inserted without difficulty.

The aspiration via the inserted NG tube revealed a 300 mL of clear yellow fluid. However, insufflations with 50 mL of air and auscultation at epigastric area were equivocal.

**Figure 1:**
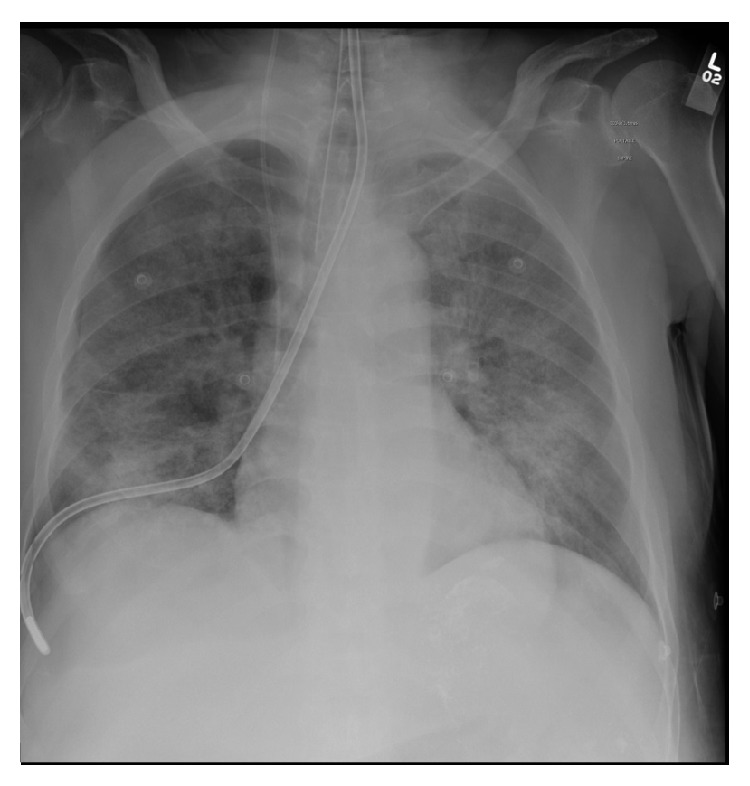


**Figure 2:**
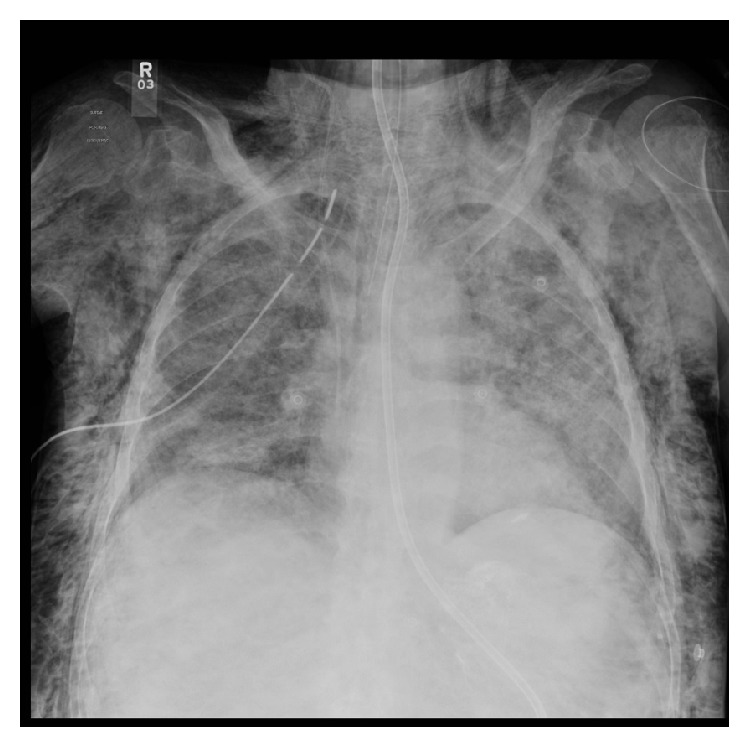


After NG tube insertion, chest X-ray showed misplaced NG tube at the right main bronchus down to the right pleural space and development of new 7.8 mm right sided pneumothorax (Figure 1, chest X-ray). The NG tube had been immediately removed and PEEP was decreased to 5 cmH_2_O. Over few minutes, the patient became progressively hypotensive and hypoxic, requiring higher doses of norepinephrine infusion and FiO_2_ of 60% to maintain 95% saturation. Right sided chest tube was inserted and the repeated X-ray revealed new subcutaneous emphysema (Figure 2). Using direct laryngoscopy technique, nasogastric tube was inserted under vision.

Over the following seven days, the patient condition had improved and the patient was successfully extubated and noninvasive ventilation was applied electively for few hours.

## 3. Discussion

Nasogastric tube (NGT) placement is a frequently performed procedure for hospitalized, particularly critically ill patients. Though it seems a simple procedure, it may carry potential life-threatening complications due to misplacement. These complications may be exacerbated by the delay in recognition or removal of misplaced tube.

In one prospective series of 740 NGT insertions in ICU patients, there was a 2% incidence of tracheopulmonary complications with a mortality of 0.3%, with pneumothoraces being the most frequent complication [1]. Other thoracic complications include erroneous bronchial placement, leading to atelectasis, pneumonia, and lung abscess (Table 1).

While the enteral nutrition is devoid of risk of complications associated with central venous catheter insertion for parental nutrition, the hazard of pulmonary complications with feeding tube insertion is comparable to that of central line [2].

Arrays of risk factors which individually or synergistically lead to NGT malposition are summarized in Table 2.

Our patient had developed an iatrogenic tension pneumothorax secondary to misplaced NG tube as a result of intricately involved potential risk factors, namely, impairment of conscious level, being critically ill, and the presence of endotracheal tube. All those factors compromise the airway reflexes, swallowing mechanism, and patient's ability to report shortness of breath or chest discomfort associated with displaced NGT. In addition, the blind insertion of NGT, stylet-stiffened feeding tube, and multiple attempts of insertion are well recognized predisposing factors of NGT complications [3, 4].

In the presented case, bedside tests were done in order to confirm appropriate positioning, starting with aspiration of gastric fluid which was falsely positive due to extraction of the yellowish pleural effusion. Then, air insufflation test was performed revealing worrisome auscultation sounds for possible tracheopulmonary insertion; for that reason, the tube was not used for feeding and a chest X-ray was requested, though it is not our routine institutional protocol, done, and confirmed a malposition NGT into the right pleural space. Criticizing the lack of the institutional protocol was addressed clearly by Weinberg and Skewes who concluded that the adoption of rigid protocols that include a mandatory radiograph immediately after the insertion of feeding tubes shows an alarming rate of 1–3% risk of feeding tubes lodging at any site in the airway down to the lung [5].

The traditional bedside techniques of gastric aspiration and insufflation test lack specificity and sensitivity and often give false reassurance that the NGT is properly positioned [6, 7].

Several suggested confirmatory tests are depicted elaborately in the literature including clinical, radiological, and laboratory investigations (Table 3).

Nevertheless, chest X-ray after insertion of feeding tube is considered a gold standard confirmatory test [6], which prevents additional complications.

In an endeavor to prevent rather than reduce the NGT insertion drawbacks, many trials and techniques had been described. In 1989, Roubenoff and Ravich proposed a two-step protocol for nasogastric tube insertion. In this procedure, the feeding tube is initially advanced blindly to 30 cm and then its position is verified by chest radiograph. After radiographic confirmation of the tube position in the esophagus, the tube is further inserted into its adequate length and a second radiograph is taken to check the final location [7].

Marderstein et al. applied that protocol at their institution and found that the rate of nasogastric tube induced pneumothorax decreased from 0.38% to 0.09%. While improving patient safety, it is a time-consuming protocol, exposing the patient to two X-rays and questioning its cost-effectiveness [3].

Additionally, the observation of the aspirate character to predict proper placement is subjective and has limited value which could be deceiving as what had occurred with our case [8].

Having inadequate conventional confirmatory methods, several new techniques are developed to overcome the misplacement and related complications. In addition to fluoroscopic and endoscopic based approaches, another device that allows for real time localization of the feeding tube tip was assessed by Young et al. with promising success rates [9]. This technology uses a signaling device at the end of the NGT which is traced by an external sensor with feedback signals as it passes through stomach, pylorus, and duodenum.

## 4. Conclusion

Airway and other significant complications rates pertinent to NGT insertion are considerable. Institutional protocol is required to reduce the substantial risk of tube misplacement of NGT.

Considering the potential life-threatening complications that may occur in case of displaced nasogastric tube, especially in critically ill patient, alternative essential confirmatory methods need to be discovered.

Capnometry method has the highest specificity and sensitivity among the other known bedside methodologies. Carbon dioxide detection monitoring may detect the respiratory displacement of the feeding tube and consequently contribute to the prevention of pulmonary complications. Nevertheless, the other techniques still aimed at early detection of anticipated adverse events rather than prevention.

Experienced operator, periprocedural risk assessment, proper technique of placement, and postprocedure confirmations are the fundamental recommendations for safe NGT insertions.

## Figures and Tables

**Table 1:** Complications of nasogastric tubes insertion.

Organ/system	Complication	
Nasopharyngeal	Hemorrhage	
	Ulceration	
	Oropharyngeal coiling	
	Eustachian tube misplacement	

Larynx	Trauma	
	Ulceration	
	Vocal cord dysfunction	
	Vocal cord paralysis	

Gastrointestinal	Coiling	Knotted tube
	Hemorrhage	Reflex esophagitis
	Ulceration	Pneumoperitoneum
	Perforation	Esophageal feeding
	Tracheoesophageal fistula	Sepsis

Pleuropulmonary (2%)	Aspiration of gastric content/enteral feed: pneumonitis, pneumonia, empyema, abscess, and sepsis Bronchial misplacement: atelectasis, collapse, pulmonary hemorrhage, and perforation	Intrapleural placement: pneumothorax (60%), haemothorax, hydrothorax, and bronchopleural fistula Airway obstruction: early or late; respiratory distress and ventilator failure

Mediastinal	Mediastinal misplacement	
	Mediastinitis	
	Pneumomediastinum	

Others	Nasogastric tube syndrome (upper airway obstruction secondary to ulceration of postcricoid region causing	
	vocal cord abduction paralysis)	
	Intracranial misplacement	
	Erosion to large vessels	

**Table 2:** Factors increasing the risk of nasogastric tube misplacement.

NGT factors	Technique factors	Patient factors
(i) Fine bore	(i) Inexperienced operator	(i) Altered mental status
(ii) Stiff monofilament core	(ii) Incorrect patient position	(ii) Critically ill patients
(iii) Stiffening wire	(iii) Blind insertion	(iii) Endotracheal intubation
(iv) Absent radiopaque marker	(iv) Incorrect NGT length	(iv) Tracheostomy
(v) Flexible polymer constriction	(v) Repeated attempts	(v) Use of sedation
	(vi) Insufficient lubricant	(vi) Use of neuromuscular blocker agents
		(vii) Anatomical facial abnormalities
		(viii) Facial trauma/inhalation injury
		(ix) Anticoagulation/thrombophilia
		(x) Upper airway/esophageal injury
		(xi) Nasopharyngeal pathology
		(xii) Following lung transplant

**Table 3:** Techniques used to confirm NG position.

The technique	Comment
Insufflation test	(i) Unreliable in small tubes or those with guide wire because of reduced airflow (ii) 20% false positive results [10, 11]

Gastric aspiration	(i) Normal gastric aspirate is clear to slightly yellow (ii) Altered in gastrointestinal bleeding and bowel obstruction

Aspirated fluid pH and bilirubin	(i) A pH less than 5 and bilirubin less than 5 mg/dL identified 98% of gastric sites (ii) A pH greater than 5 and bilirubin less than 5 mg/dL identified 100% of the respiratory sites [12]

Capnometry	Reported high specificity and sensitivity rate [13, 14]

Capnography	Capnography was as accurate as colorimetric device for detecting CO_2_ during placement of NG tubes [15]

Magnetic guidance	(i) Relatively new technique (ii) Rule out the presence of the NGT in stomach and lung

## References

[1] Rassias A. J., Ball P. A., Corwin H. L. (1998). A prospective study of tracheopulmonary complications associated with the placement of narrow-bore enteral feeding tubes. *Critical Care*.

[2] Al-Jahdali H., Irion K. L., Allen C., de Godoy D. M., Khan A. N. (2012). Imaging review of procedural and periprocedural complications of central venous lines, percutaneous intrathoracic drains, and nasogastric tubes. *Pulmonary Medicine*.

[3] Marderstein E. L., Simmons R. L., Ochoa J. B. (2004). Patient safety: effect of institutional protocols on adverse events related to feeding tube placement in the critically ill. *Journal of the American College of Surgeons*.

[4] Wang P.-C., Tseng G.-Y., Yang H.-B., Chou K.-C., Chen C.-H. (2008). Inadvertent tracheobronchial placement of feeding tube in a mechanically ventilated patient. *Journal of the Chinese Medical Association*.

[5] Weinberg L., Skewes D. (2006). Pneumothorax from intrapleural placement of a nasogastric tube. *Anaesthesia and Intensive Care*.

[6] Pillai J. B., Vegas A., Brister S. (2005). Thoracic complications of nasogastric tube: review of safe practice. *Interactive Cardiovascular and Thoracic Surgery*.

[7] Roubenoff R., Ravich W. J. (1989). Pneumothorax due to nasogastric feeding tubes: report of four cases, review of the literature, and recommendations for prevention. *Archives of Internal Medicine*.

[8] Metheny N. A., Schnelker R., McGinnis J. (2005). Indicators of tube site during feeding. *Journal of Neuroscience Nursing*.

[9] Young R. J., Chapman M. J., Fraser R., Vozzo R., Chorley D. P., Creed S. (2005). A novel technique for post-pyloric feeding tube placement in critically ill patients: a pilot study. *Anaesthesia & Intensive Care*.

[10] Benya R., Langer S., Mobarhan S. (1990). Flexible nasogastric feeding tube tip malposition immediately after placement. *Journal of Parenteral and Enteral Nutrition*.

[11] Metheny N. A., Smith L., Stewart B. J. (2000). Development of a reliable and valid bedside test for bilirubin and its utility for improving prediction of feeding tube location. *Nursing Research*.

[12] Araujo-Preza C. E., Melhado M. E., Gutierrez F. J., Maniatis T., Castellano M. A. (2002). Use of capnometry to verify feeding tube placement. *Critical Care Medicine*.

[13] Joanna Briggs Institute (2010). Methods for determining the correct nasogastric tube placement after insertion in adults. *Best Practice*.

[14] Burns S. M., Carpenter R., Truwit J. D. (2001). Report on the development of a procedure to prevent placement of feeding tubes into the lungs using end-tidal CO_2_ measurements. *Critical Care Medicine*.

[15] Burns S. M., Carpenter R., Blevins C. (2006). Detection of inadvertent airway intubation during gastric tube insertion: capnography versus a colorimetric carbon dioxide detector. *American Journal of Critical Care*.

